# The Role of Conformational Preorganization in the Reactivity of *cis*‐1,2‐Dimesylate‐bis(benzyloxy)cyclooctane: An Activation Strain Perspective

**DOI:** 10.1002/open.70251

**Published:** 2026-06-30

**Authors:** Selçuk Eşsiz, Emine Salamci

**Affiliations:** ^1^ Department of Medical Services and Techniques Vocational School of Health Services Hakkari University Hakkari Türkiye; ^2^ Department of Chemistry Faculty of Sciences Atatürk University Erzurum Türkiye

**Keywords:** activation strain model analysis, aminocyclitols, azide, bis(benzyloxy)cyclooctane‐1,2‐diyl dimethanesulfonate, noncovalent interaction analysis

## Abstract

A computational study was performed to elucidate the factors governing the reactivity of a *cis*‐substituted cyclooctane dimesylate toward sodium azide. The calculated energy profile reveals a clear kinetic preference for formation of the monosubstituted intermediate, while the second S_N_2 substitution is associated with a significantly higher activation barrier. Activation Strain Model (ASM) analysis indicates that this increase arises primarily from a larger distortion energy and less favorable interaction energy. Consistent with these findings, Non–Covalent Interaction (NCI) analysis reveals diminished stabilizing interactions and enhanced steric repulsion in the higher‐energy transition state. Further analysis of the cyclooctane conformations demonstrates that the two pathways differ in their torsional distortion patterns. The lower‐energy transition state proceeds through a more preorganized geometry, whereas the higher‐energy pathway requires additional conformational reorganization to achieve the reactive arrangement of the leaving group. Together, these results establish conformational preorganization as a key factor governing reactivity in this flexible ring system.

## Graphical abstract

Selective monosubstitution in cyclooctane dimesylates is investigated by DFT calculations. Despite favorable SN2 conditions, the second substitution is suppressed due to increased activation strain and unfavorable conformational reorganization. The results reveal that intrinsic structural constraints, rather than external factors, govern reactivity, providing mechanistic insight into the origin of this pronounced kinetic selectivity.

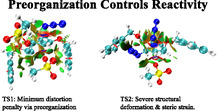

## 1 Introduction

Aminocyclitols, which are polyhydroxylated amino cycloalkanes, constitute an important class of natural and synthetic products that exhibit diverse biological activities [1, 3]. Some synthetic azidocyclitols, which serve as precursors to aminocyclitols and triazoles, have been shown to possess potent anticancer properties [4, 6]. Moreover, organic azides are commonly encountered in the structures of pharma­ceuticals and other bioactive molecules [7, 8]. Motivated by these features, we have recently developed a range of synthetic methodologies for C8‐azidocyclitols, which serve as valuable precursors for the synthesis of C8‐aminocyclitols [1, 2, 9, 11].

**SCHEME 1:**
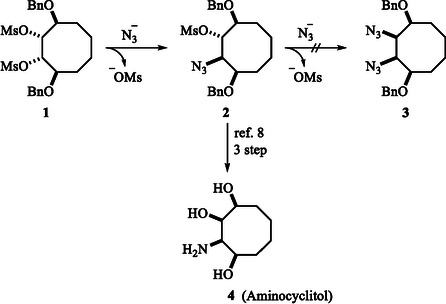
Stepwise azide substitution of dimesylate **1** [10].

In a recent experimental study, we attempted to synthesize diaminocyclooctanediol via nucleophilic substitution of dimesylate **1** with sodium azide; however, only one mesylate group underwent substitution to afford the corresponding monoazide [10]. In the present work, we investigate the mechanism of this transformation using density functional theory (DFT). The reaction of dimesylate **1** with azide is proposed to proceed through a stepwise S_N_2 substitution pathway (Scheme 1). Experimentally, when the reaction was carried out in DMF at 105°C, the diazide product **3** was not detected; instead, monoazide **2** was isolated in 83% yield after 4 days [10]. These results indicate a pronounced kinetic preference for the first substitution step, while the second substitution is significantly less favorable under the same conditions. Although this exclusive monosubstitution was observed experimentally, the underlying origin of the pronounced kinetic selectivity remained unresolved in our previous work [10]. In particular, the relative contributions of conformational effects, steric constraints, and electronic factors have not yet been fully elucidated. To rationalize this behavior at the molecular level, we present a detailed DFT study. By computing activation free energies and performing Non–Covalent Interaction (NCI) and Activation Strain Model (ASM) analyses [12], we aim to elucidate the electronic and structural factors governing the kinetic barriers of the individual substitution steps.

## 2 Results and Discussion

**FIGURE 1:**
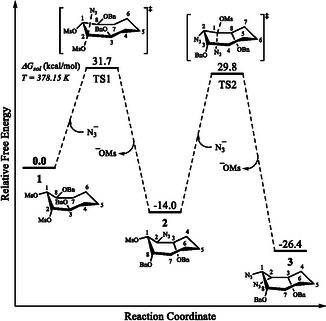
Relative free energy profile for stepwise azide substitution.

The reaction mechanism was investigated using DFT, focusing on the nucleophilic substitution pathway. The results for the reaction of *cis*‐substituted cyclooctane dimesylate **1** with sodium azide are presented in Figure 1. In the first step, nucleophilic attack of the azide ion (N_3_^−^) at the C2 carbon (corner position) of the cyclooctane framework proceeds via transition state TS1, leading to the formation of the monoazido derivative **2**. For this step, reaction and activation free energies are −14.0 and 31.7 kcal mol^−1^, respectively. Following the formation of the monoazido derivative **2**, the second nucleophilic substitution was examined. In Its lowest‐energy conformation, the remaining mesylate group is not ideally oriented for backside attack. Therefore, prior to the second substitution, a conformational reorganization is required to place the leaving group in a pseudo‐axial orientation compatible with an S_N_2 mechanism. This conformational equilibrium is not explicitly depicted in the simplified reaction profile but introduces an additional energetic penalty. As a result, transition state TS2 is accessed from a higher‐energy conformer of monoazide **2**, leading to a significantly increased activation barrier of 43.8 kcal mol^−1^. Despite the second substitution being thermodynamically favorable (ΔG = −12.4 kcal mol^−1^), the substantial kinetic barrier effectively prevents its occurrence under the experimental conditions. These computational findings are in full agreement with the experimental observations, which indicate that the reaction terminates at the monoazide stage without proceeding to the disubstituted product.

**FIGURE 2:**
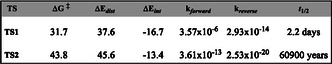
Calculated activation free energies (ΔG^‡^, kcal mol^−1^), distortion energies (Δ*E*_dist_, kcal mol^−1^), interaction energies (Δ*E*_int_, kcal mol^−1^), rate constants (*k*, *s* ^−1^), and theoretical half‐lives (*t*_1/2_) for the substitution steps at 378.15 K.

**FIGURE 3:**
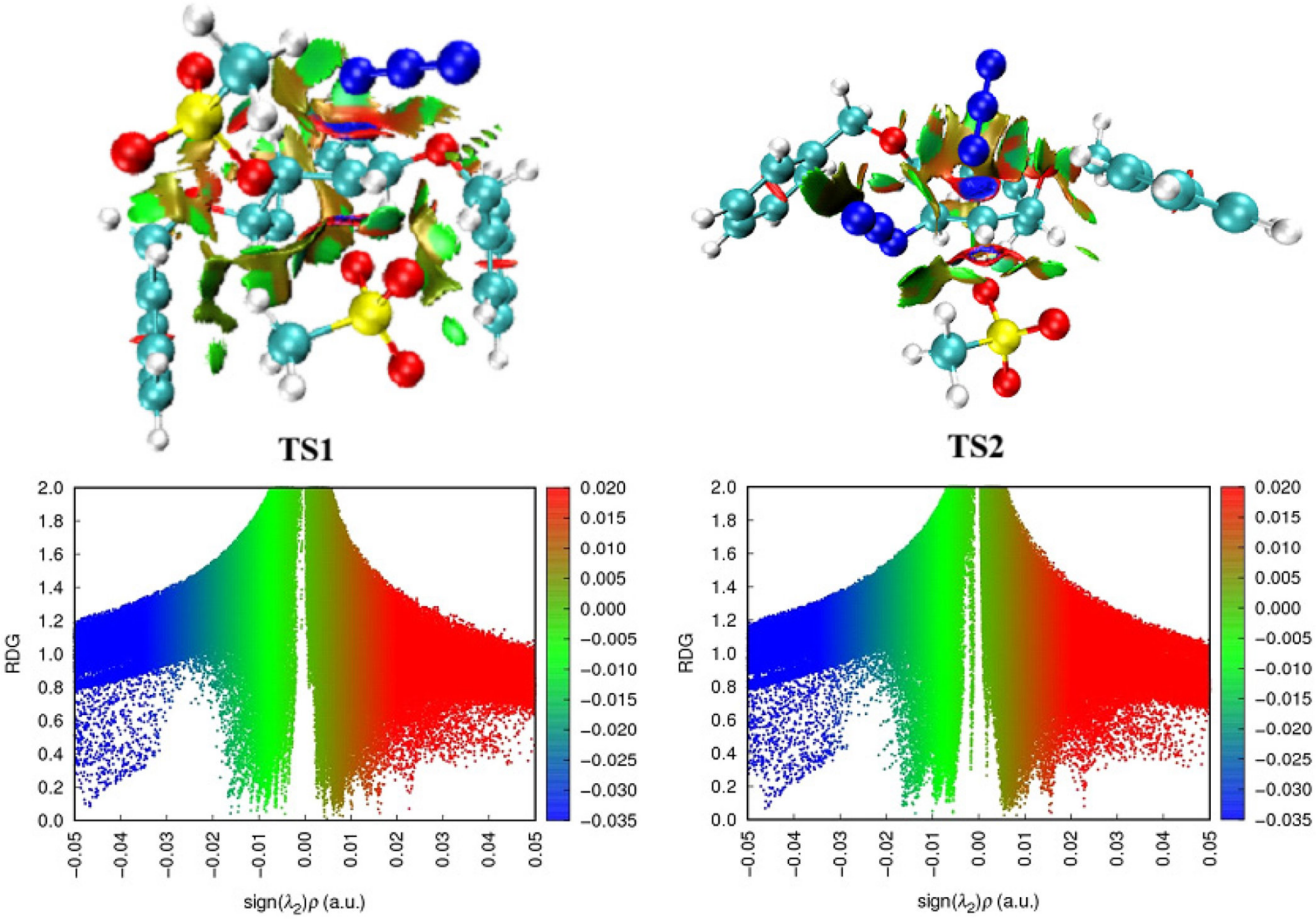
Origin of reactivity differences: NCI analysis of transition states.

The calculated activation parameters and kinetic data for both substitution steps are summarized in Figure 2. A significant increase in the activation free energy was observed when moving from the first substitution to the second, with the barrier rising by 12.1 kcal mol^−1^. To further understand the origin of this substantial energetic penalty, an ASM combined with NCI plots was performed (Figure 3).

The NCI analysis reveals that the benzene rings in TS1 are strategically positioned to wrap around the molecular core, facilitating stabilizing dispersive interactions (indicated by the extensive green surfaces) that lead to a more favorable interaction energy (−16.7 kcal mol^−1^). In contrast, the benzene rings in TS2 are forced into more distant positions to accommodate the conformational requirements of the second substitution (quasi‐equatorial to quasi‐axial reorganization). In addition, the TS2 geometry introduces unfavorable gauche interactions between the newly installed azide group and the adjacent OBn substituent, as well as increased electrostatic repulsion between the two azide groups. This loss of molecular compactness in TS2 not only diminishes the stabilizing dispersive interactions but also exacerbates transannular interactions between the hydrogen atoms of the cyclooctane framework. These coupled effects translate into a significant 8.0 kcal mol^−1^ increase in distortion energy (Δ*E*_dist_), which in turn accounts for the prohibitively high activation barrier of the second transition state. Consequently, the synergy between increased structural strain and diminished stabilization leads to the observed kinetic divergence. This is reflected in the computed half‐lives (*t*_1/2_), where the first step proceeds in 2.2 days (consistent with the 4‐day experimental observation), whereas the second step remains effectively suppressed with a half‐life of 60 900 years, providing a robust explanation for why the reaction selectively halts at the monoazide stage and fails to yield the di‐substituted product.

**FIGURE 4:**
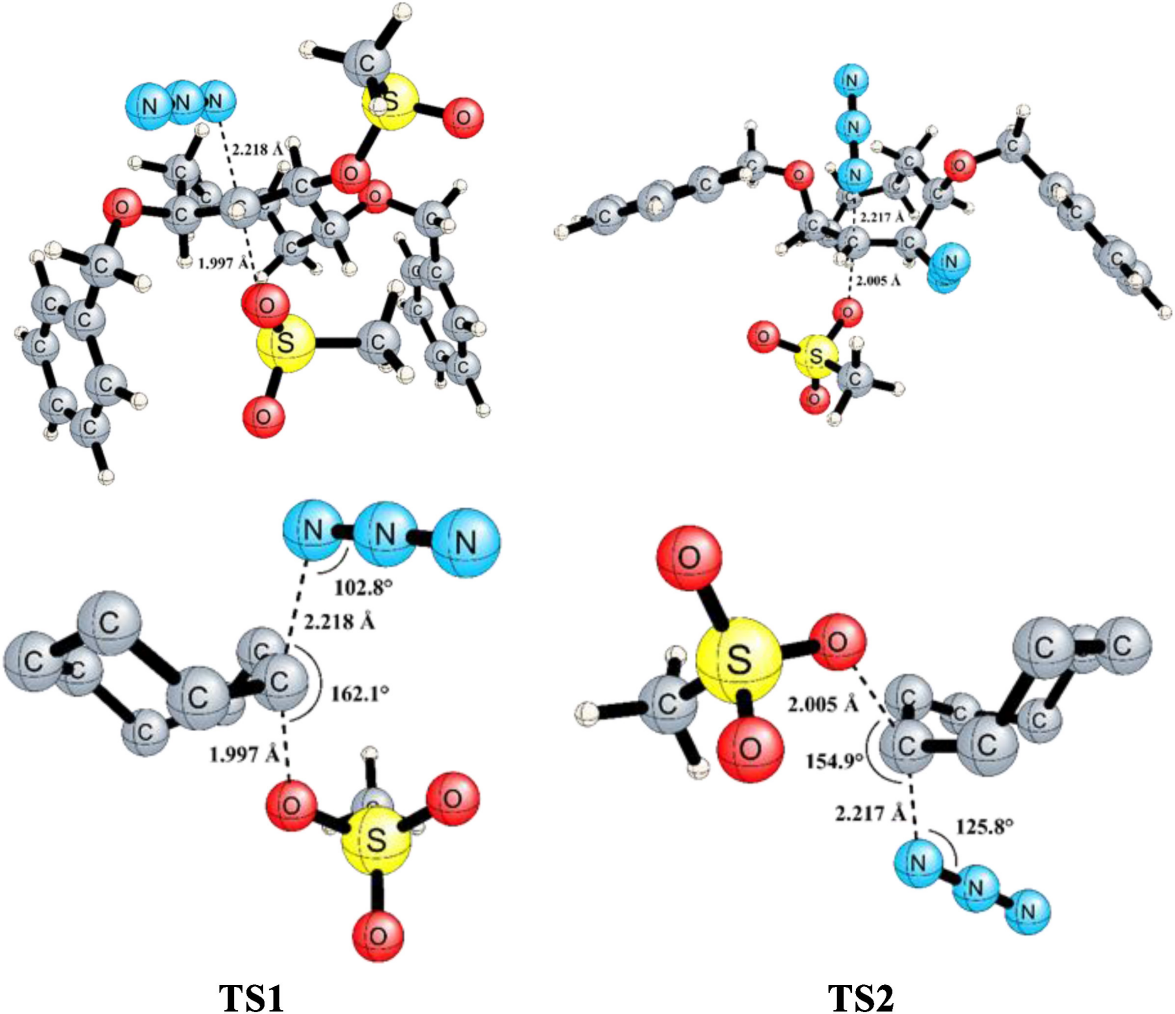
Optimized geometries of transition states TS1 and TS2 with selected bond lengths (Å) and angles (°).

Geometric analysis of the transition states reveals a pronounced change in the nucleophile’s approach trajectory between the two steps (Figure 4). In TS1, the reaction proceeds with a relatively favorable N‐C‐O angle of 162.1° and a more flattened C–N–N approach angle of 102.8°, which is closer to the ideal tetrahedral angle, and thus enables a smoother, less hindered entry into the reaction center. In contrast, TS2 exhibits significant angular distortion, with the N‐C‐O angle reduced to 154.9^°^ and a more head‐on trajectory defined by a C‐N‐N angle of 125.8^°^. This substantial deviation from the preferred sp^3^ geometry forces the azide into a sterically congested region, thereby contributing to the observed kinetic quenching. Compared to TS1, the less favorable approach trajectory in TS2 contributes to a higher activation barrier.

Compared to the forward rate constants, the reverse rate constants were found to be several orders of magnitude smaller (Figure 2). Therefore, the backward reactions were neglected, and the system was treated as effectively irreversible under the studied conditions. The resulting kinetic model is shown below.

Based on this model, the time‐dependent concentration changes of the species can be described by the following differential Equations (1)–(3)

(1) $$\frac{d[1]}{dt}=-k_{1}\left[1\right]$$

(2) $$\frac{d[2]}{dt}=k_{1}\left[1\right]-k_{2}\left[2\right]$$

(3) $$\frac{d[3]}{dt}=k_{2}\left[2\right]$$

The analytical solutions to Equations (1)–(3), under the condition *k*_1_ ≠ *k*_2_ are given in Equations (4)–(6)

(4) $$\left[1\right]\left(t\right)={\left[1\right]}_{0}e^{-k_{1}t}$$

(5) $$\left[2\right]\left(t\right)={\left[1\right]}_{0}\frac{k_{1}}{k_{2}-k_{1}}\left(e^{-k_{1}t}-e^{-k_{2}t}\right)$$

(6) $$\left[3\right]\left(t\right)={\left[1\right]}_{0}\left[1-\frac{k_{2}e^{-k_{1}t}-k_{1}e^{-k_{2}t}}{k_{2}-k_{1}}\right]$$

**FIGURE 5:**
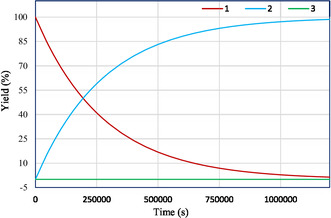
Time‐dependent concentration profiles for the azide substitution of dimesylate **1** at 378.15 K.

The reaction progress over time was modeled using the calculated rate constants, as illustrated in Figure 5. The kinetic profile clearly shows the rapid consumption of dimesylate **1** (red line) and the concomitant formation of monoazido intermediate **2** (blue line), reaching nearly complete conversion within the simulated timeframe. Notably, the yield of the di‐azide product **3** (green line) remains negligible throughout the process, even at extended time intervals. This behavior underscores the high selectivity of the reaction and confirms that the extreme activation barrier of the second step effectively prevents any further substitution, maintaining the system at the monoazide stage. The time‐dependent concentration profiles were obtained by evaluating the analytical solutions of the rate Equations (4)–(6) using the calculated rate constants, and the resulting kinetic curves were generated using Microsoft Excel.

The strong temperature dependence of the rate constants further supports this interpretation. Although the reaction is conducted at elevated temperature (378.15 K), which facilitates overcoming the moderate barrier associated with the first substitution step, the substantially higher barrier of the second step remains kinetically inaccessible even under these conditions. In addition, the use of a polar aprotic solvent such as DMF is expected to enhance the nucleophilicity of the azide ion and generally promote SN2 processes. However, the persistence of complete selectivity toward monosubstitution under these favorable conditions indicates that the reaction outcome is not limited by solvent effects but rather governed by intrinsic structural factors. In particular, the conformational rigidity and steric constraints of the cyclooctane framework impose a significant energetic penalty on the second substitution, preventing its occurrence despite otherwise favorable reaction conditions.

## 3 Conclusions

In summary, this study provides a detailed mechanistic rationale for the divergent reactivity observed in the double nucleophilic substitution of a *cis*‐1,2‐dimesylate cyclooctane **1**. The computed energy profile establishes a clear kinetic preference for the first substitution step (TS1), which proceeds with an activation barrier of 31.7 kcal mol^−1^, whereas the second substitution (TS2) exhibits a substantially higher barrier of 43.8 kcal mol^−1^, identifying it as the rate‐limiting step of the overall transformation.

Distortion‐interaction analysis reveals that this increase is primarily driven by a significant rise in distortion energy (37.6 vs 45.6 kcal mol^−1^), indicating that the cyclooctane framework must undergo considerably greater geometric deformation to access the second transition state. This effect is further reinforced by a less favorable interaction energy (−16.7 vs −13.4 kcal mol^−1^), consistent with NCI analysis showing increased steric repulsion and diminished stabilizing interactions.

Compared with the lower‐energy pathway, the higher barrier in TS2 can be attributed to the lack of preorganization of the OMs leaving group. In the TS1 pathway, nucleophilic attack occurs at an OMs group already positioned at the reactive corner site, enabling a direct S_N_2 displacement with minimal conformational adjustment. By contrast, TS2 requires relocation of the OMs group from the head region to the corner position prior to bond formation, introducing additional torsional distortion and conformational reorganization. Torsional analysis supports this interpretation by showing that the lower‐energy transition state involves a more localized and cooperative adjustment of dihedral angles, whereas the higher‐energy structure exhibits a more distributed and nonuniform distortion pattern. Collectively, these results indicate that conformational preorganization, rather than bond formation alone, is the decisive factor governing reactivity in this flexible ring system. This behavior may be general for medium‐sized rings exhibiting head‐to‐corner reorganization penalties.

## 4 Experimental Section

### 4.1 Computational Methods

Conformational sampling was performed using the CREST program (version 3.0.2) using default settings [13], which employ the iMTD‐GC algorithm at the GFN2‐xTB level of theory with an energy window of 6.0 kcal mol^−1^ [14]. The ten lowest‐energy conformers were subsequently reoptimized in the solution phase (SMD/DMF) at the M06‐2X/6‐311++G(d, p) level of theory using Gaussian 16 [15, 20]. Frequency calculations confirmed the structures as local minima. The transition states were confirmed by intrinsic reaction coordinate (IRC) computations (see the Supporting Information for details) [21, 22]. The IRC analyses indicate that each transition state smoothly connects the corresponding reactant and product minima without the presence of additional stationary points along the reaction pathways. Thermochemical corrections were applied using the GoodVibes v3.2 program to match the experimental conditions (378.15 K, 0.162 M) [23]. To account for the limitations of the harmonic oscillator approximation, Truhlar’s quasi‐harmonic correction was employed with a frequency cut‐off of 100 cm^−1^ [24]. Enthalpic contributions were further refined using the Head‐Gordon approximation [25]. A vibrational scaling factor of 0.970 was applied for calculations at the M06‐2X/6‐311++G(d, p) level of theory.

Noncovalent interaction (NCI) analysis was performed using the reduced density gradient (RDG) approach to investigate and characterize weak intra‐ and intermolecular interactions. The calculations were carried out with the Multiwfn (version 2026.4.10) program [26, 27], and the resulting RDG isosurfaces were visualized using VMD (version 2.0.0a7) [28]. The NCI plots were generated by mapping the electron density (*ρ*) and the sign of the second eigenvalue of the electron density Hessian matrix [sign(*λ*_2_)*ρ*] onto the isosurfaces. This method allows for the visual differentiation of interaction types based on a standard color scale: blue regions indicate strong attractive interactions (such as hydrogen bonding), green areas represent weak van der Waals forces, and red regions signify strong steric repulsion. The isosurfaces were plotted at an RDG value of 0.5 a.u. to highlight regions of significant noncovalent interactions. Additionally, the open‐source software cheMVP.exe was used for 3D structure visualization.

## Author Contributions

**Selçuk Eşsiz**: conceptualization, investigation, methodology, software, formal analysis, visualization, writing – original draft, writing – review and editing. **Emine Salamci**: writing – review and editing, writing – original draft, validation, formal analysis.

## Conflicts of Interest

The authors declare no conflicts of interest.

## Supporting information

Supplementary material

## Data Availability

The data underlying this study are available in the published article and its online Supporting Information.

## References

[1] E. Salamci , “Synthetic Strategies for Aminocyclitols: An Updated Review,” Tetrahedron 189 (2026): 134988.

[2] E. Salamci , “Recent Developments Concerned with the Synthesis of Aminocyclitols,” Tetrahedron Letters 61 (2020): 151728.

[3] A. Delgado , “Recent Advances in the Chemistry of Aminocyclitols,” European Journal of Organic Chemistry 23 (2008): 3893–3906.

[4] K. M. Sureshan , K. Ikeda , N. Asano , and Y. Watanabe , “Efficient Syntheses of Optically Pure Chiro‐ and Allo‐Inositol Derivatives, Azidocyclitols and Aminocyclitols from Myo‐Inositol,” Tetrahedron 64 (2008): 4072–4080.

[5] G. Brunn , A. H. Fauq , S. Chow , A. P. Kozikowski , A. Gallegos , and G. Powis , “Cellular Pharmacology of D‐3‐Azido‐3‐Deoxy‐Myo‐Inositol, an Inhibitor of Phosphatidylinositol Signaling Having Antiproliferative Activity,” Cancer Chemotherapy and Pharmacology 35 (1994): 71–79. 7987980 10.1007/BF00686287

[6] G. Powis , I. A. Aksoy , D. C. Melder , et al., “D‐3‐Deoxy‐3‐Substituted Myo‐Inositol Analogues as Inhibitors of Cell Growth,” Cancer Chemotherapy and Pharmacology 29 (1991): 95–104. 1760864 10.1007/BF00687317

[7] S. Brase and K. Banert , Organic Azides: Syntheses and Applications (Wiley‐VCH Weinheim, 2010).

[8] Y. Durust , H. Karakus , M. Kaiser , and D. Tasdemir , “Synthesis and *anti*‐Protozoal Activity of Novel *D*ihydropyrrolo [3,4‐D][1,2,3] Triazoles,” European Journal of Medicinal Chemistry 48 (2012): 296–304. 22217867 10.1016/j.ejmech.2011.12.028

[9] I. Polat , S. Essiz , U. Bozkaya , and E. Salamci , “Efficient and Regioselective Synthesis of Dihydroxy‐Substituted 2‐Aminocyclooctane‐1‐Carboxylic Acid and Its Bicyclic Derivatives,” Beilstein Journal of Organic Chemistry 18 (2022): 77–85. 35047084 10.3762/bjoc.18.7 PMC8744459

[10] E. Salamci and A. K. Lafzi , “Efficient Synthesis of Aziridinecyclooctanediol and 3‐Aminocyclooctanetriol,” Beilstein Journal of Organic Chemistry 18 (2022): 1539–1543. 36447522 10.3762/bjoc.18.163 PMC9663974

[11] I. Polat , S. Essiz , and E. Salamci , “Experimental and DFT Studies on the Regioselective Methanolysis of 5‐Azido‐9‐Oxabicyclo [6.1. 0] Nonan‐4‐Yl 4‐Nitrobenzoate Isomers,” Beilstein Journal of Organic Chemistry 22 (2026): 547–556. 41929661 10.3762/bjoc.22.40 PMC13040264

[12] F. M. Bickelhaupt and K. N. Houk , “Analyzing Reaction Rates with the Distortion/Interaction‐Activation Strain Model,” Angewandte Chemie International Edition 56 (2017): 10070–10086. 28447369 10.1002/anie.201701486 PMC5601271

[13] P. Pracht , F. Bohle , and S. Grimme , “Automated Exploration of the Low‐Energy Chemical Space with Fast Quantum Chemical Methods,” Physical Chemistry Chemical Physics 22 (2020): 7169–7192. 32073075 10.1039/c9cp06869d

[14] C. Bannwarth , S. Ehlert , and S. Grimme , “GFN2‐xTB‐An Accurate and Broadly Parametrized Self‐Consistent Tight‐Binding Quantum Chemical Method with Multipole Electrostatics and Density‐Dependent Dispersion Contributions,” Journal of Chemical Theory and Computation 15 (2019): 1652–1671. 30741547 10.1021/acs.jctc.8b01176

[15] A. V. Marenich , C. J. Cramer , and D. G. Truhlar , “Universal Solvation Model Based on Solute Electron Density and on a Continuum Model of the Solvent Defined by the Bulk Dielectric Constant and Atomic Surface Tensions,” The Journal of Physical Chemistry B 113 (2009): 6378–6396. 19366259 10.1021/jp810292n

[16] Y. Zhao and D. G. Truhlar , “The M06 Suite of Density Functionals for Main Group Thermochemistry, Thermochemical Kinetics, Noncovalent Interactions, Excited States, and Transition Elements: Two New Functionals and Systematic Testing of Four M06‐Class Functionals and 12 Other Functionals,” Theoretical Chemistry Accounts 120 (2008): 215–241.

[17] R. Krishnan , J. S. Binkley , R. Seeger , and J. A. Pople , “Self‐Consistent Molecular Orbital Methods. XX. A Basis Set for Correlated Wave Functions,” Journal of Chemical Physics 72 (1980): 650–654.

[18] A. D. McLean and G. S. Chandler , “Contracted Gaussian Basis Sets for Molecular Calculations. I. Second Row Atoms, Z = 11‐18,” Journal of Chemical Physics 72 (1980): 5639–5648.

[19] T. Clark , J. Chandrasekhar , G. W. Spitznagel , and P. V. R. Schleyer , “Efficient Diffuse Function‐Augmented Basis Sets for Anion Calculations. III. The 3‐21+G Basis Set for First‐Row Elements, Li‐F,” Journal of Computational Chemistry 4 (1983): 294–301.

[20] M. J. Frisch , G. W. Trucks , H. B. Schlegel , et al., Gaussian 16, Revision C.01 (Gaussian, Inc, 2019).

[21] K. Fukui , “Formulation of the Reaction Coordinate,” Journal of Physical Chemistry 74 (1970): 4161–4163.

[22] K. Fukui , “The Path of Chemical Reactions ‐ the IRC Approach,” Accounts of Chemical Research 14 (1981): 363–368.

[23] G. Luchini , J. V. Alegre‐Requena , I. Funes‐Ardoiz , and R. S. Paton , “GoodVibes: Automated Thermodynamic Corrections for Solution‐Phase Computational Chemistry,” F1000Research 9 (2020): 291.

[24] R. F. Ribeiro , A. V. Marenich , C. J. Cramer , and D. G. Truhlar , “Use of Solution‐Phase Vibrational Frequencies in Continuum Solvent Models,” Journal of Physical Chemistry B 115 (2011): 14556–14562. 21875126 10.1021/jp205508z

[25] Y. Li , J. Gomes , S. M. Sharada , A. T. Bell , and M. Head‐Gordon , “Improved Combined Internal Energy and Entropy Scale for the Characterization of Adsorption and Reaction in Zeolites,” Journal of Physical Chemistry C 119 (2015): 1840–1850.

[26] T. Lu and F. Chen , “Multiwfn: A Multifunctional Wavefunction Analyzer,” Journal of Computational Chemistry 33 (2012): 580–592. 22162017 10.1002/jcc.22885

[27] T. Lu , “Multiwfn: A Comprehensive Analyzer for Chemical Wavefunctions,” Journal of Chemical Physics 161 (2024): 082503. 39189657 10.1063/5.0216272

[28] W. Humphrey , A. Dalke , and K. Schulten , “VMD: Visual Molecular Dynamics,” Journal of Molecular Graphics 14 (1996): 33–38. 8744570 10.1016/0263-7855(96)00018-5

