# Supplementary material for: The Role of Conformational Preorganization in the Reactivity of cis‐1,2‐Dimesylate‐bis(benzyloxy)cyclooctane: An Activation Strain Perspective
Source: ChemistryOpen. 2026 Jun 30;15(7):e70251. doi: 10.1002/open.70251 (PMC13318916; doi:10.1002/open.70251)
Supplement: Supplementary file 1 — Supplementary material [file OPEN-15-e70251-s001.pdf]

The Role of Conformational Preorganization in the Reactivity of *cis*-Cyclooctane Dimesylate: An Activation Strain Perspective

Selçuk Eşsiz* and Emine Salamci

Department of Medical Services and Techniques, Vocational School of Health Services, Hakkari University, 30000 Hakkari, Türkiye

Dpartment of Chemistry, Faculty of Science, Atatürk University, 25240 Erzurum, Türkiye

Contents

1. IRC Pathways S1

2. Cartesian Coordinates for Optimized Structures S2

1. IRC Pathways

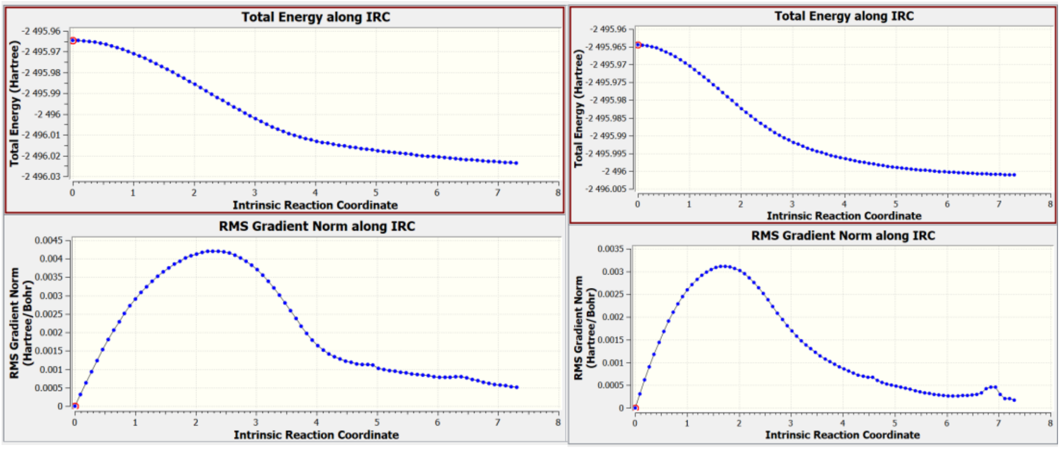

Figure S1. Composite IRC profile for TS1. The forward (left) and reverse (right) pathways are combined to illustrate the full minimum energy path (MEP) from reactants to products.

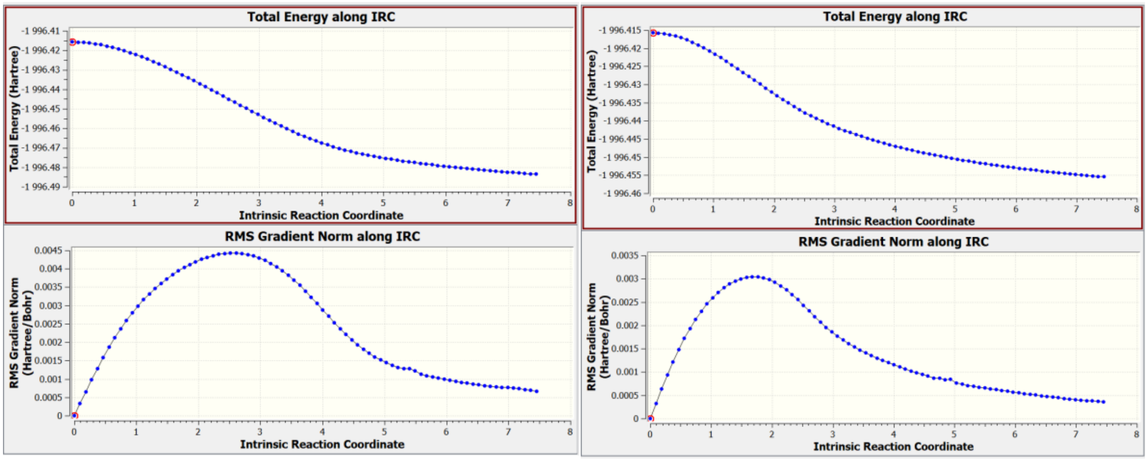

Figure S2. Composite IRC profile for TS1. The forward (left) and reverse (right) pathways are combined to illustrate the full minimum energy path (MEP) from reactants to products.

2. Cartesian Coordinates for Optimized Structures

1

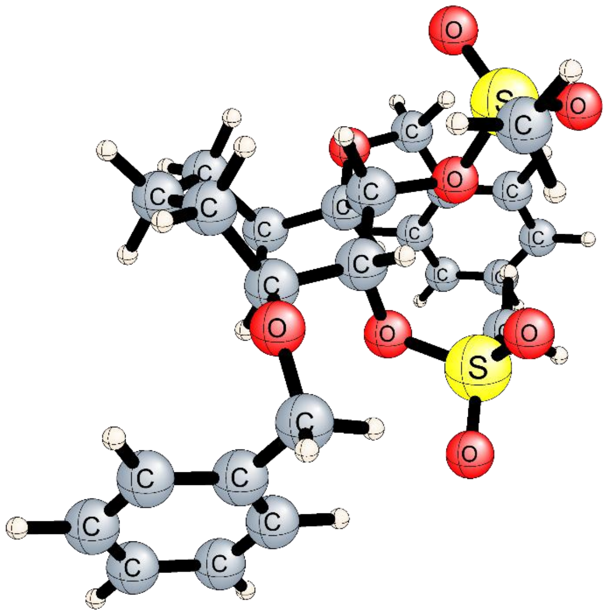

Zero-point correction= 0.546037 (Hartree/Particle)

Thermal correction to Energy= 0.578277

Thermal correction to Enthalpy= 0.579221

Thermal correction to Gibbs Free Energy= 0.480627

Sum of electronic and zero-point Energies= -2331.144562

Sum of electronic and thermal Energies= -2331.112322

Sum of electronic and thermal Enthalpies= -2331.111378

Sum of electronic and thermal Free Energies= -2331.209972

E= -2331.6905991 a.u., number of negative frequencies = 0

0 1

C	-0.60590500	-1.17137300	0.43663300
C	0.67581800	-0.87313800	-0.34231700
C	2.03692300	-1.28877200	0.25240900
C	2.00305500	-2.49882200	1.19353400
C	1.93281400	-2.15455700	2.68501200
C	0.62409900	-1.56438700	3.21422000
C	0.24131500	-0.19103300	2.62924500
C	-0.89435100	-0.23716200	1.61226200
O	-2.04014100	-0.65498200	2.33997900

C	-3.55839800	1.04090600	1.36015800
C	-4.29567600	1.26934700	0.19975500
C	-4.52796200	2.56918700	-0.24800100
C	-4.00784200	3.65111000	0.45507900
C	-3.26506000	3.42944100	1.61514200
C	-3.04831200	2.13246500	2.06785600
C	-3.34726100	-0.37179600	1.85054000
C	4.33828400	0.34640900	-0.65401400
C	4.13063500	1.72404400	-0.63880500
O	2.91256400	-1.63599800	-0.81743200
C	4.91825000	2.54696000	0.16646300
C	5.90882100	1.99346000	0.97101200
C	6.11333400	0.61327900	0.96800700
C	5.33336800	-0.20443500	0.15810600
C	3.49885600	-0.55347600	-1.53171800
O	0.74253800	0.56098900	-0.58600900
C	-1.31896400	2.00540700	-1.41429900
S	0.18066500	1.23411600	-1.94517700
O	-0.09026800	0.18241200	-2.91053200
O	1.14993400	2.25491900	-2.28790600
O	-1.69774700	-0.95842100	-0.50625300
C	-1.36456400	-2.82500000	-2.38781500
S	-2.48710400	-2.19832500	-1.17391000
O	-3.62369000	-1.57647300	-1.81765400
O	-2.70963600	-3.20441800	-0.15316600
H	-0.62449900	-2.21169700	0.76546200
H	0.60158100	-1.37691100	-1.30720000
H	2.45601900	-0.43112500	0.78727500
H	2.94744700	-3.01906300	1.02048100
H	1.21147900	-3.20085300	0.91292500
H	2.14095500	-3.07242600	3.24273300
H	2.75117500	-1.46038900	2.91194000
H	0.72567500	-1.46703400	4.29781400
H	-0.19513400	-2.27302100	3.05400100

H	-0.11218100	0.46681500	3.42597900
H	1.10971100	0.30728700	2.19082300
H	-1.05078000	0.76656500	1.20668300
H	-4.67138700	0.42293200	-0.36713200
H	-5.10146300	2.73355700	-1.15364100
H	-4.17648100	4.66258500	0.10283400
H	-2.85757200	4.26915100	2.16717100
H	-2.46980000	1.96077300	2.97088000
H	-3.99550000	-0.55416600	2.71245000
H	-3.64386200	-1.07880100	1.07337000
H	3.34153200	2.14906300	-1.25136900
H	4.75064100	3.61833400	0.17050800
H	6.51890500	2.63136500	1.60067400
H	6.88415500	0.17794800	1.59429300
H	5.49117600	-1.27861000	0.15437600
H	2.74616300	0.03848100	-2.06069900
H	4.12840200	-1.03066200	-2.28623600
H	-2.01263000	1.22262100	-1.11807700
H	-1.07648000	2.67511500	-0.58859300
H	-1.69604800	2.56631300	-2.27096700
H	-1.91367000	-3.58297100	-2.94819800
H	-1.06414100	-1.99785500	-3.02985700
H	-0.51646500	-3.27855600	-1.87429200

2

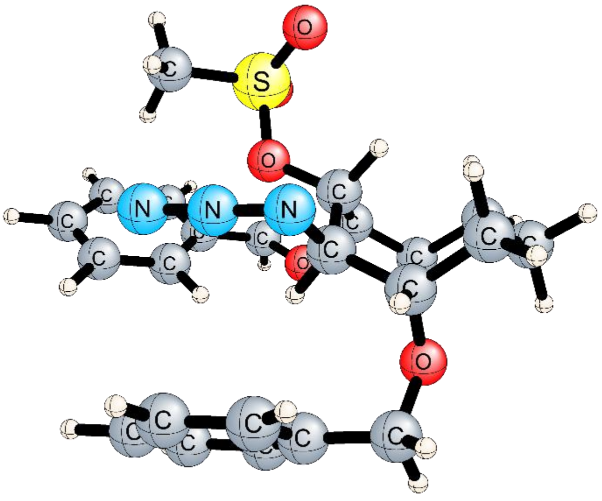

Zero-point correction= 0.506275 (Hartree/Particle)

Thermal correction to Energy= 0.535498

Thermal correction to Enthalpy= 0.536442

Thermal correction to Gibbs Free Energy= 0.445927

Sum of electronic and zero-point Energies= -1831.653703

Sum of electronic and thermal Energies= -1831.624480

Sum of electronic and thermal Enthalpies= -1831.623536

Sum of electronic and thermal Free Energies= -1831.714050

E= -1832.1599777 a.u., number of negative frequencies = 0

0 1

C	0.25626100	1.48264900	-0.18043200
C	1.23740500	0.38658600	-0.61980700
C	2.75669900	0.72855900	-0.61236100
C	3.13715300	2.20017000	-0.84168400
C	3.32240500	3.09372300	0.39070500
C	2.09344000	3.43853400	1.23538700
C	1.46234000	2.23659700	1.95968800
C	0.17020200	1.74532200	1.32511200
O	-0.24847700	0.56657200	1.99959200
C	-2.39492300	-0.56961800	1.60642400
N	0.93340100	0.03238200	-2.03542200
N	0.07130000	-0.82827300	-2.17251400
N	-0.69379600	-1.62154200	-2.39448400
C	-3.24376800	0.74643200	-1.81939800
S	-2.18581800	1.97139700	-1.11470000
O	-1.04687500	0.97561000	-0.57801100
C	-3.78933900	-0.55689700	1.68258200
C	-4.54441600	-1.52867800	1.03155200
C	-3.91079800	-2.52468800	0.29030800
C	-2.52015300	-2.53963100	0.20935800
C	-1.76753000	-1.56850800	0.86590500
C	-1.61715400	0.48531700	2.36197300
O	3.40249100	0.24274000	0.55492400

C	2.99160000	-2.14842900	0.20393900
C	2.24190600	-2.61655700	1.28607500
C	1.25105800	-3.57462600	1.09693700
C	0.99928800	-4.07536000	-0.18026000
C	1.74879800	-3.62181500	-1.26238000
C	2.74150900	-2.66252600	-1.06866600
C	4.00178900	-1.04325100	0.40613200
H	0.45063400	2.40618500	-0.72963500
H	1.07873000	-0.48744500	0.01526400
H	3.14650200	0.17179100	-1.46980300
H	2.42445500	2.64573000	-1.54444700
H	4.09938800	2.18547500	-1.36126500
H	4.06811300	2.62693200	1.04103200
H	3.76332600	4.02990400	0.03445800
H	1.34446600	3.95227500	0.62081800
H	2.41218200	4.16861100	1.98355300
H	2.17094100	1.40741300	2.00905300
H	1.20910100	2.50364100	2.98830900
H	-0.59098600	2.52462000	1.45038600
O	-1.62079500	2.83370200	-2.13797000
O	-2.83389200	2.61234900	0.01684300
H	-2.72827600	0.29562200	-2.66593000
H	-3.47285900	0.01398200	-1.04433300
H	-4.28637700	0.22370900	2.25162300
H	-5.62671200	-1.50398300	1.09611300
H	-4.49625900	-3.27950500	-0.22250800
H	-2.01690800	-3.30663200	-0.37020400
H	-0.68606200	-1.57675200	0.79370000
H	-1.65432300	0.24393000	3.43041900
H	-2.10923500	1.45331600	2.22762500
H	2.43175700	-2.21815500	2.27847800
H	0.67123700	-3.92874700	1.94234000
H	0.22663000	-4.82160800	-0.32847100
H	1.56003300	-4.01179900	-2.25648200

H	3.32347400	-2.30501600	-1.91289800
H	4.70516900	-1.02238500	-0.43428400
H	4.56208000	-1.20589300	1.32830800
H	-4.14570400	1.26684900	-2.14464900

3

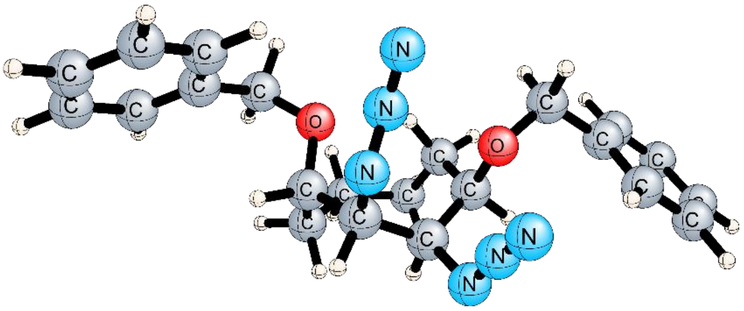

Zero-point correction= 0.467512 (Hartree/Particle)

Thermal correction to Energy= 0.493707

Thermal correction to Enthalpy= 0.494651

Thermal correction to Gibbs Free Energy= 0.408452

Sum of electronic and zero-point Energies= -1332.160833

Sum of electronic and thermal Energies= -1332.134638

Sum of electronic and thermal Enthalpies= -1332.133694

Sum of electronic and thermal Free Energies= -1332.219892

E= -1332.6283447 a.u., number of negative frequencies = 0

0 1

C	0.68670300	0.10974600	1.53203200
C	-0.78890800	-0.30109600	1.33724100
C	-1.81692600	0.75563000	0.88615200
C	-1.62262900	2.15188100	1.50694000
C	-0.82117800	3.17315600	0.68818100
C	0.68165500	2.95463700	0.47809300
C	1.05665100	1.76033300	-0.41902900
C	1.55125400	0.53756400	0.34906600
O	1.67725600	-0.61479000	-0.47125700
C	4.01529000	-0.25850400	-1.06888700
C	4.75152600	0.81073400	-1.57369100
C	6.05084100	1.04934800	-1.12550200

C	6.61611400	0.22385300	-0.15937300
C	5.88102800	-0.84414800	0.35646200
C	4.58932500	-1.08348200	-0.09736500
C	2.60558900	-0.52823400	-1.54268000
N	1.38852800	-0.95136500	2.29960000
N	1.54259300	-2.04416200	1.75965800
O	-1.87265300	0.78651000	-0.53013700
N	1.75661400	-3.08972300	1.40758900
N	-0.96863600	-1.57649500	0.61280600
N	-0.81018100	-1.60673700	-0.60511700
N	-0.69662100	-1.80041000	-1.70414100
C	-4.26907700	0.37741100	-0.77218200
C	-5.41202000	0.85041000	-0.13071700
C	-6.48701000	-0.00163600	0.12104600
C	-6.41926800	-1.33778800	-0.25965300
C	-5.27587500	-1.81950000	-0.89741400
C	-4.20894300	-0.96566200	-1.15362600
C	-3.09749900	1.29323000	-1.04690900
H	0.66997400	0.94282200	2.23365900
H	-1.10238300	-0.55978800	2.35074200
H	-2.77212400	0.35945500	1.25083700
H	-1.19669200	2.03731000	2.50937500
H	-2.61842200	2.57915500	1.65879800
H	-1.28852200	3.27770600	-0.29652800
H	-0.94781900	4.13830700	1.18854900
H	1.19304500	2.88280900	1.44515200
H	1.06518700	3.86576800	0.01200400
H	0.20905100	1.47216800	-1.04487300
H	1.86780300	2.06126300	-1.08718200
H	2.53157000	0.78089500	0.78332700
H	4.30607200	1.46065600	-2.32075600
H	6.61617400	1.88296700	-1.52699300
H	7.62442100	0.40961900	0.19321000
H	6.31854200	-1.49017800	1.10949500

H	4.01614800	-1.91522900	0.30186800
H	2.29836800	0.23179600	-2.26782000
H	2.55383300	-1.50203600	-2.03526300
H	-5.45994600	1.89070900	0.17665100
H	-7.37148600	0.37777000	0.62049400
H	-7.25168900	-2.00339100	-0.06093700
H	-5.21995500	-2.85985800	-1.19794500
H	-3.32270000	-1.33892700	-1.65769400
H	-2.93569100	1.38820900	-2.12314500
H	-3.30551800	2.29120800	-0.64576300

TS1

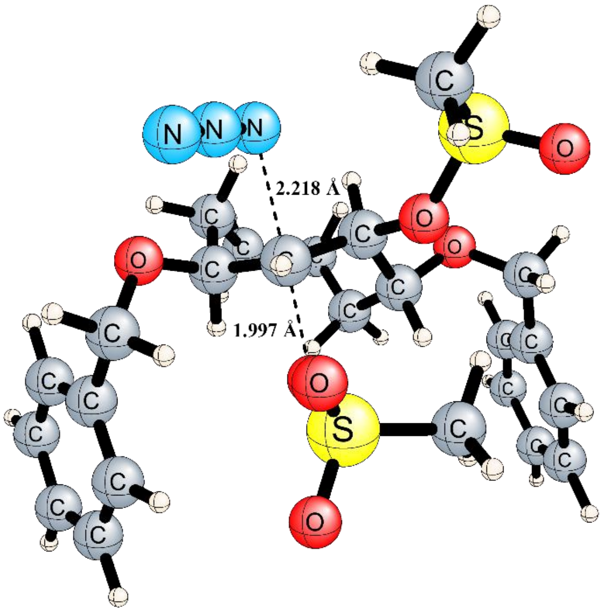

Zero-point correction= 0.555892 (Hartree/Particle)

Thermal correction to Energy= 0.591966

Thermal correction to Enthalpy= 0.592910

Thermal correction to Gibbs Free Energy= 0.484162

Sum of electronic and zero-point Energies= -2495.408554

Sum of electronic and thermal Energies= -2495.372480

Sum of electronic and thermal Enthalpies= -2495.371536

Sum of electronic and thermal Free Energies= -2495.480284

E= -2495.964446 a.u., im. freq. = 606.06*i* cm^{-1}

0 1

C	-0.65373800	1.04666600	-0.60543000
C	1.88238200	0.50476800	-1.14788600
C	1.62559900	0.72810100	-2.64858600
C	1.02143200	-0.45736500	-3.40518200
C	-0.45134600	-0.76872900	-3.12694300
C	-0.73758100	-1.28755600	-1.70651200
C	-1.39587000	-0.28783100	-0.76454500
O	-2.68818500	0.06223000	-1.25267600
C	-3.98219500	-1.72535100	-0.19808000
C	-4.00566800	-2.31007600	1.06656400
C	-4.22438400	-3.68018700	1.21158400
C	-4.41626800	-4.47572600	0.08709400
C	-4.39310000	-3.89781200	-1.18295900
C	-4.17800600	-2.53169400	-1.32294400
C	-3.74937100	-0.23926100	-0.35646000
C	4.26586400	-0.81283300	0.12216900
C	4.08001800	-1.79608500	1.09212200
O	3.11850000	1.11977200	-0.82138800
C	4.58046300	-3.08306200	0.89671000
C	5.25899000	-3.39861100	-0.27656100
C	5.43574500	-2.42212500	-1.25666400
C	4.94294600	-1.13694800	-1.05668600
C	3.75303400	0.59470200	0.33535000
O	0.73550200	-0.59362200	0.95000500
C	-1.08557100	-0.57360400	2.84902600
S	0.64913100	-0.45615700	2.46560500
O	1.11103400	0.87396600	2.89133600
O	1.31193500	-1.58847800	3.11869900
O	-1.34787100	1.77780900	0.42846400
C	-1.40339700	4.34445900	0.95713100
S	-2.26969300	3.06349100	0.09688900
O	-3.53165900	2.82335400	0.76903100
O	-2.27246600	3.31635700	-1.32943900
H	-0.74671000	1.59344500	-1.54503000

H	1.96312100	-0.57254700	-0.96608000
H	2.61246900	0.93212700	-3.07046000
H	1.02916600	1.62838300	-2.80588200
H	1.12888000	-0.24639300	-4.47361900
H	1.61974100	-1.35412300	-3.20255900
H	-0.77105600	-1.52600100	-3.84731100
H	-1.06265400	0.11730300	-3.33253000
H	-1.42682200	-2.13397500	-1.75342900
H	0.17470300	-1.66411900	-1.23797300
H	-1.49189800	-0.74631400	0.21937800
H	-3.85100000	-1.68886500	1.94380600
H	-4.23879900	-4.12346900	2.20111500
H	-4.58357900	-5.54144500	0.19600300
H	-4.54480500	-4.51464300	-2.06186300
H	-4.15494500	-2.08165400	-2.31078600
H	-4.63391100	0.23815800	-0.78506100
H	-3.56221500	0.21530300	0.62186100
H	3.52075400	-1.56246800	1.99272900
H	4.42949300	-3.84135600	1.65735600
H	5.64359900	-4.40054400	-0.43130100
H	5.96045100	-2.66352800	-2.17458400
H	5.07761700	-0.37637300	-1.81924800
H	3.09002900	0.62761000	1.20647600
H	4.58840200	1.27190300	0.53122600
H	-1.60583800	0.24662400	2.35500900
H	-1.44416200	-1.54118200	2.49816700
H	-1.18837700	-0.49902300	3.93184900
H	-2.04891000	5.22315900	0.93423000
H	-1.24146700	4.01134000	1.98159400
H	-0.46781900	4.52833900	0.43445800
H	1.08628700	1.52922700	0.69554700
C	0.80041400	1.02656400	-0.21564000
N	2.54817900	3.98318200	0.65502200
N	1.80882400	3.56796900	-0.13193300

N	1.03622200	3.12088200	-0.90813100

TS2

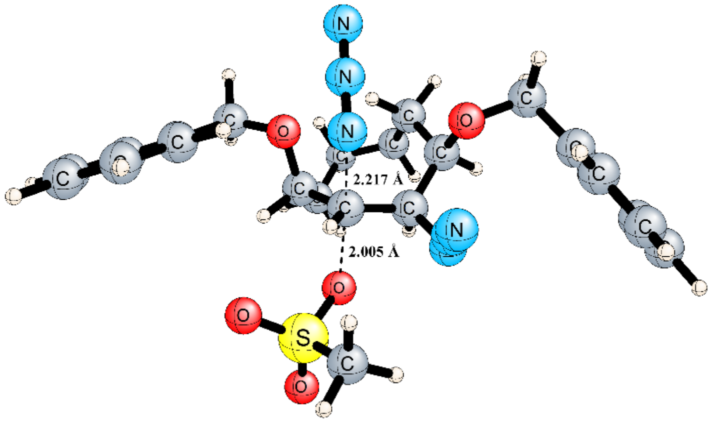

Zero-point correction= 0.516944 (Hartree/Particle)

Thermal correction to Energy= 0.549788

Thermal correction to Enthalpy= 0.550732

Thermal correction to Gibbs Free Energy= 0.449954

Sum of electronic and zero-point Energies= -1995.898815

Sum of electronic and thermal Energies= -1995.865970

Sum of electronic and thermal Enthalpies= -1995.865026

Sum of electronic and thermal Free Energies= -1995.965805

E= -1996.4157585 a.u., im. freq. = 598.74*i* cm^{-1}

0 1

C	-1.06249700	0.13743100	0.65359300
C	1.56382600	-0.42154500	0.97843500
C	1.36024600	-0.61053100	2.50021600
C	0.80351500	-1.95757500	2.96668700
C	-0.68086300	-2.21460900	2.69967100
C	-1.05034800	-2.38770400	1.21444500
C	-1.81961800	-1.20903200	0.63078600
O	-2.28915200	-1.49960100	-0.67404200
C	-4.64442400	-0.91459400	-0.53779600
C	-5.34737400	-0.74056500	0.65412000
C	-6.24217900	0.31845400	0.80153200
C	-6.43792300	1.21420400	-0.24503700
C	-5.74710600	1.04154400	-1.44458400

C	-4.86026700	-0.01940000	-1.58913100
C	-3.61859200	-2.01235700	-0.69443500
N	-1.89237400	1.17292500	0.00884400
N	-1.89590100	1.17926000	-1.21931700
O	1.91730200	-1.64499300	0.35865500
N	-1.95993300	1.32856600	-2.33061200
N	0.24604400	-0.89043600	-1.66717500
N	0.30481000	-2.06298000	-1.77388300
N	0.36568000	-3.21238100	-1.90214700
C	4.22849800	-1.21031500	-0.28503300
C	5.53782200	-0.99354100	0.14343200
C	6.43819600	-0.29883000	-0.66100100
C	6.03081800	0.19536200	-1.89764200
C	4.72041600	-0.00983800	-2.32500400
C	3.82332400	-0.71132200	-1.52417200
C	3.26716200	-2.01105800	0.56745600
H	-1.05988400	0.42901100	1.70448400
H	2.37837200	0.29476900	0.83426000
H	0.75371800	0.20802100	2.88911400
H	2.34717000	-0.48437900	2.95530600
H	1.38989900	-2.77073500	2.52629000
H	0.96689500	-2.00889300	4.04789600
H	-1.27487000	-1.40582600	3.14424000
H	-0.96191500	-3.12227300	3.23997100
H	-0.15754100	-2.57803000	0.61640600
H	-1.70283300	-3.25772000	1.09597000
H	-2.68625400	-1.01491000	1.27615300
H	-5.19223100	-1.43767700	1.47199000
H	-6.78196400	0.44462000	1.73344800
H	-7.13112400	2.04020300	-0.13155800
H	-5.90527300	1.73129200	-2.26638200
H	-4.32474300	-0.15741600	-2.52332100
H	-3.74946500	-2.77145000	0.08532600
H	-3.72986500	-2.49763300	-1.66590600

H	5.85316200	-1.36793100	1.11285400
H	7.45383800	-0.13547000	-0.31783100
H	6.72839700	0.74242000	-2.52187700
H	4.39598200	0.37922400	-3.28401300
H	2.79604800	-0.86100500	-1.84394300
H	3.32463000	-3.06785800	0.29071300
H	3.54782500	-1.93122200	1.62422700
H	0.64731500	0.86916600	-0.60550900
C	0.38983800	0.24706200	0.23086500
O	0.59309700	1.90134500	1.34472800
S	1.30762900	3.10659400	0.75178900
C	0.21579100	3.72841000	-0.51316000
H	0.64983100	4.65432600	-0.89079000
H	0.15158900	2.99740700	-1.31948200
H	-0.76174000	3.91371100	-0.06974900
O	2.55112500	2.69819600	0.08298100
O	1.44326800	4.15732400	1.76363300
